# Supplementary material for: Pet Owners’ Preferences for Quality of Life Improvements and Costs Related to Innovative Therapies in Feline Pain Associated with Osteoarthritis—A Quantitative Survey
Source: Animals (Basel). 2024 Aug 8;14(16):2308. doi: 10.3390/ani14162308 (PMC11350694; doi:10.3390/ani14162308)
Supplement: Supplementary file 1 [file animals-14-02308-s001.zip › animals-3057510-supplementary.pdf]

Supplementary Material

1.1 Development phase: interviews with pet owners

1.1.1 Methodology

Three semi-structured 60-minute interviews were conducted with the owners of cats with confirmed OA. Each interview covered a variety of topics, including background information of each cat, their diagnosis, and treatment experience. These identified relevant signs and behaviors of OA in cats that are concerning to pet owners and important to address with therapy. The interviews included both a spontaneous elicitation exploratory component and a probed component that focused on an array of QoL.

1.1.2 Results

Outputs from the qualitative interviews with pet owners highlight that the domains listed in (**Table S1**) served as key QoL attributes when considering pet owner decision-making in the context of pain associated with OA.

Draft QoL descriptions were developed to explore how impactful each individual attribute is when considering pet owners WTP. Draft QoL descriptions at the development stage are presented in **Table S2**.

Confidential - Not for Public Consumption or Distribution

1.2 Feedback phase: veterinarian interviews

1.2.1 Methodology

Three semi-structured 60-minute interviews were conducted with veterinarians with experience in treating cats with pain associated with OA. Each interview covered a variety of topics, including a general overview of how veterinarians would describe pain associated with OA, the diagnosis process, preferred treatment regimens, and reactions to QoL descriptions. The veterinarian interviews featured a subsection that focused on their reactions to the QoL descriptions previously developed from pet owner interviews.

1.2.2 Results

Veterinarians' responses to the attributes identified by pet owners suggested the following: inclusion of "stiffness" when referring to walking/gait, removal of vocalization, and addition of facial expression in pain expression; the addition of eating less due to poor mobility for appetite, and inclusion of willingness to explore outside for outdoor cats (**Table S3**).

When presented with the draft QoL descriptions from the development phase of this research, all veterinarians stated that the QoL descriptions were accurate reflections of the impairment associated with OA. Minor amendments were suggested such as, the removal of cats "frequently" walking with a limp within the mobility domain, the addition of facial expression changes within pain expression, and the addition of a difference in coat appearance within well-being. The revised QoL descriptions, following feedback from the veterinarians, were taken forward for use in the WTP survey design and are presented in **Table S4**.

1.3 WTP survey design

This section presents introductory and consent form wording. Also, see **Table 5** for an overview of the survey structure and content.

1.3.1 Introductory wording from WTP survey

- We are conducting research to understand the aspects that are most important to cat owners, when it comes to treating osteoarthritis in cats.

- If you choose to participate, you will be asked to complete an online survey.

- The survey is expected to last approximately 45 minutes.

- The survey includes questions about your personal preference between the situations and treatments that may affect your cat.

- As part of the survey, we will also ask you some additional questions about yourself and your cat.

- If you are interested in participating, please click below, and we will ask you some questions to determine if you are eligible to participate.

☐ I am interested in participating in this survey and agree to answer some questions to determine if I am eligible to participate.

1.3.2 Informed consent form from WTP survey

Introduction

Thank you for agreeing to participate in this online survey. We are recruiting on behalf of Adelphi Values, a research agency acting on behalf of a pharmaceutical company. Adelphi Values is totally independent as a company and would like you to be completely honest with your views. Everything you say will be treated in total confidence.

The purpose of this questionnaire is to understand the aspects that are most important to cat owners, when it comes to treating pain associated with osteoarthritis in cats. The outputs of this research will be used to identify the pet owners' willingness-to-pay to help veterinarians overcome price objections for a new treatment.

The questionnaire will be an online survey which will take approximately 45 minutes to complete. Your responses will automatically save as you proceed, therefore you may close and re-join the questionnaire at any point. Upon completion of this questionnaire, you will receive an incentive in appreciation of your time and cooperation. This will have been confirmed in a separate notification. Once again, we would like to stress the importance in your honesty in answering this survey based on your personal opinions and experience.

We will comply with all national laws protecting your personal data and with relevant guidelines including Insights Association, PMRG, BHBIA, MRS, ESOMAR, EphMRA and all other relevant national codes of practice. The aim of this research is to gain your views and is not intended to be promotional and no one will try to sell you anything. All information provided will remain confidential and will only be reported as group data with no identifying information.

- I confirm that I am happy to take part in this survey voluntarily and know that I may terminate the survey or withhold information if I so wish.
- I understand that all data from this survey will only be used for research purposes.
- I understand that anything I see or read during this research should be treated as confidential. The research presented should not be used to influence decisions outside of this research project.

Please click here to view Adelphi Values statement regarding data protection, privacy, invisible processing and ways to contact Adelphi Values: http://www.adelphigroup.com/privacypolicy.pdf

You can contact Adelphi Values at: *[compliance contact email address redacted for publication]*

You can find out more about Adelphi Values at https://www.adelphigroup.com/adelphi-values/

Adverse Event reporting

It is a requirement that we pass on to our client details of adverse events that are raised within the surveys. Although this is an online research survey and how you respond will, of course, be treated in confidence, should you raise an adverse events and / or product complaints, we will need to report this, even if it has already been reported by you directly to the company or the regulatory authority. The Adverse Event data collected may be sent outside the reporting country for processing.

We will initially forward the Adverse Report to the pharmaceutical company's pharmacovigilance department anonymously. Any requests for further information from pharmacovigilance will come through the recruiting agency.

☐ Yes, I agree to allow my name and contact information to be released, only in conjunction with an adverse event

☐ No, I do not agree to have my name or contact information released in conjunction with an adverse event; I wish to remain anonymous

Your participation and renumeration in this research are NOT dependent upon your response.

For all respondents (present on new page)

The research will comply with UK Data Protection law and with the British Healthcare Business Intelligence Association's Legal & Ethical Guidelines.

You will remain anonymous unless you give permission to be identified. Your responses will be added to the answers of other participants to provide an overall understanding. Your information will only be used for this purpose and will not be passed to any third party without your permission. If you wish to see it, we will be happy to supply a copy of the privacy policy that applies to your participation in this research.

We may want to further explore your responses to some of the questions answered. Would you be prepared to be re-contacted to take part in any follow-up research on this topic?

☐ Yes

☐ No

By proceeding to the next screen, I confirm that I have read understood and accept the statements presented in the previous screens and I am happy to proceed with the survey on this basis.

☐ YES, I am happy to proceed with the survey on this basis

☐ NO, I am not happy to proceed with the survey on this basis and I do not wish to continue.

Supplementary Tables and Figures

Table S1. Prioritized attributes following pet owner interviews.

Mobility	Pain expression	Well-being
> Jumping<br>> Walking/gait<br>> Running	> Tolerance to touch<br>> Vocalization	> Appetite<br>> Sleeping habits.

Table S2. QoL descriptions following development interviews with pet owners

Domain	Healthy cat	Cat suffering from pain associated with OA
**Mobility**	Your cat is able to move around freely, and appears to be as energetic and active as you may typically expect	Your cat may often hesitate before jumping up and down from surfaces, may sometimes appear uncomfortable and stiff, and may frequently walk with a limp.
**Pain expression**	You are able to stroke and comfort your cat as you normally would without your cat reacting to your touch, or vocalizing pain upon movement.	Your cat may frequently appear to be in pain when your stroke particular areas (such as their back legs) and may vocalize pain by hissing or meowing in response to touch.
**Well-being**	Your cat appears to be "themselves" and does not appear to have any changes in sleeping habits, toileting, or appetite.	The cat may demonstrate changes in their sleeping habits, or appetite and may demonstrate changes in toileting (such as, no longer toileting outside if an outdoor cat, or missing the litter box sometimes, spraying urine outside of the box due to difficulty in maintaining their posture while urinating).

OA: Osteoarthritis.

Table S3. Prioritized attributes following veterinarian interviews.

Mobility	Pain expression	Well-being
> Jumping<br>> Walking/gait (stiffness)<br>> Running	> Tolerance to touch<br>> Facial expression	> Appetite (eating less due to poor mobility)<br>> Sleeping habits<br>> Willingness to explore outside.

Table S4. QoL descriptions following the interviews with veterinarians

Domain	Healthy cat	Cat suffering from pain associated with OA
**Mobility**	Your cat is able to move around freely, and appears to be as energetic and active as you may typically expect	Your cat may often hesitate before jumping up and down from surfaces, may sometimes appear uncomfortable and stiff, and may walk with a limp.
**Pain expression**	You are able to stroke and comfort your cat as you normally would without your cat reacting to your touch, or vocalizing pain upon movement.	Your cat may frequently appear to be in pain when your stroke particular areas (such as their back legs) **and may appear to be in pain (noticed in facial expression) when touched.**
**Well-being**	Your cat appears to be "themselves" and does not appear to have any changes in sleeping habits, toileting, or appetite.	The cat may demonstrate changes in their sleeping or **grooming habits (their coat may look different/ appear matted)** or appetite, or may demonstrate changes in toileting (such as, no longer toileting outside if an outdoor cat, or missing the litter box sometimes, spraying urine outside of the box due to difficulty in maintaining their posture while urinating).

Table S5. Overview of WTP survey design.

Screening questions, further questions on demographic and pet details, and introductory information	
↓	
Presentation of QoL descriptions: A healthy cat vs a cat suffering from pain associated with OA	— Providing context for the QoL descriptions to be tested later within the survey design
↓	
×15 direct trade-off questions on improving domains of QoL	— testing which domains respondents prefer to improve
×1 test trade-off question with a clearly inferior answer	— confirming respondents' understanding of the trade-offs
↓	
×3 questions on WTP to improve domains of QoL with therapy	— testing which domains respondents would pay to improve, and how much
↓	
×8 statements for respondents to provide their level of agreement with	— testing respondents' agreement regarding product attributes
↓	
×1 question exploring pet owner preferences across product profiles (product A, SoC vs product B, mAb)	— testing preferences between therapies and how much more they would be WTP for the novel mAb (no cost presented)
x1 rating scale exercise on product attribute importance	—in terms of administration, efficacy and safety
x3 rating scale exercise on willingness to initiate therapies	— testing willingness to initiate therapy for any therapy, SoC and novel mAb
x1 exploring pet owner preferences across full product profiles (product A, SoC vs product B, mAb)	— testing preferences between therapies WTP (costs presented)
↓	
×1 question on preference between veterinarian and veterinarian nurse administration of product B	— testing if respondents are comfortable with injectable therapies delivered by veterinarian nurses
↓	
Conclusion and contact information	
